# Bringing classes back into poverty discussions

**DOI:** 10.3389/fsoc.2022.969750

**Published:** 2023-01-17

**Authors:** Gizem Özgün, Antoine Dolcerocca

**Affiliations:** ^1^Department of Social Policy, Middle East Technical University, Ankara, Türkiye; ^2^Department of Sociology, Middle East Technical University, Ankara, Türkiye

**Keywords:** poverty, absolute poverty, relative poverty, capability approach, development, social exclusion, neoliberalism, capital-labor relations

## Abstract

The 1980s saw a shift in the emphasis of discourse on poverty from production relations to consumption relations, with levels of consumption and purchasing power used to define poverty. Based on this concept, much of the research establishes absolute poverty lines or develops relative indicators to distinguish between “poor” and “non-poor.” This paper makes the case that such poverty measurement, while useful for assessing trends over the long term or taking into account relative dynamics, distorts our knowledge of poverty by hiding its root causes and results in overly optimistic interpretations. These discussions also decontextualize poverty from its political and economic context by uncritically accepting and promoting neoliberal regime. Moreover, the article questions the meaning of the “eradication of poverty” and suggests that the nominal rise in PPP income obscures historical capitalist accumulation processes (such as dispossession, proletarianization and depeasantization). As a result, the study suggests to recenter the analysis on the material causes of poverty, which are rooted in the functioning of the capitalist system, its antagonistic character, and the class-based contradictions of production itself.

## Introduction

Most discussions on poverty start off by trying to measure poverty and equate measuring poverty with defining it and evaluating its dynamics (Rowntree, 1902; Townsend, 1979; Bradshaw and Mayhew, 2011; Ferreira et al., 2015). Instead of understanding the causes of poverty, these approaches start by measuring poverty, and the measurement itself defines poverty. But should poverty be defined as an income threshold or as falling short of meeting certain consumption levels? Can we define poverty simply as being excluded or relatively poor compared to others? Do such approaches allow us to understand the causes of poverty and propose adequate solutions? We argue that mainstream perspectives on poverty today tend to offer partial explanations and we suggest to recenter the analysis on the material causes of poverty, which are rooted in the functioning of the capitalist system, its antagonistic and polarizing character, and the class-based contradictions of production itself. The purpose of this paper is to present a Marxist critique on poverty discussions grounded in the antagonistic and polarizing character of capitalist accumulation, classes, and the contradictions of production relations. The article is organized in the following way: we start with a critical review of the main definitions of poverty, from absolute and relative poverty, to the capability, human development, and social exclusion approaches. Then, in a second section, we discuss the limitations of these mainstream approaches and propose to recenter poverty analysis on class relations.

## Mainstream approaches to poverty

### Absolute poverty

The absolute poverty approach is a monetary measurement of poverty based on consumption or income level of individual and/or household in purchasing power parity.^1^ Such approach to defining and measuring poverty was pioneered by Rowntree (1902) and Townsend (1979). His study, which he conducted in 1899 in York with “wage-earning classes,” is considered the first instance of the absolute poverty approach. In the study, he sought to analyze poverty's depth and extent by creating a poverty line calculated with the cash-equivalent of the average nutritional needs of adults and children in addition to clothing, fuel, household sundries, and rent. This total sum was defined as the “minimum sum necessary” for the “maintenance of physical efficiency,” and poverty under this poverty line was described as “primary poverty” (Rowntree, 1902).

As already evident from this early example, the absolute poverty concept is a way of defining the basic needs of individuals and their cash equivalents to calculate poverty lines which help to differentiate poor from non-poor by identifying those who fail to meet a certain consumption and/or income level. It therefore requires determining the necessary subsistence for individuals' survival (in Rowntree's case, nutrition-intake calculations) and aims to solving poverty by meeting the basic needs of individuals.

About a century after this early absolute poverty study, we observe a sharp rise in interest in this approach, spearheaded by International Financial Organizations, such as the World Bank, in the 1990s. The World Bank has been one of the leading supporters of this approach since the 1990s, especially under the rubric of extreme poverty calculations for “undeveloped countries” and has been using poverty thresholds to differentiate poor from non-poor with consumption-based analysis. This approach consists in defining individuals and households' absolute minimum needs, defining policies to help them meet those needs and therefore reduce consumption-based poverty.

This absolute poverty line approach is explained in the 1990 Development Report (World Bank, 1990) and supported by the research of Ravallion et al. (1991). It uses the 1 dollar per person and per day threshold (in 1985 PPP) as absolute poverty line for the poorest countries. This absolute poverty line, called “extreme poverty line,” has changed through time (according to the changes in cost of living throughout the world) from $1 to $1.08 (in 1993 PPP), $1.25 (in 2005 PPP), and $1.90 (in 2011 PPP) (Ferreira et al., 2015; World Bank, 2021). Consequently, individuals with income below these lines have been qualified as living in extreme poverty. These measures have also been used to reach Millennium Development goals (later reframed as Sustainable Development Goals) by the United Nations, namely, halving the portion of people whose income is < $1.25 per day between 1990 and 2015 and eradicating extreme poverty by 2030, i.e., have no individuals whose income is under $1.90 per day by 2030 (Ferreira et al., 2016).

Although this approach is frequently used in the literature (Ravallion et al., 1991; Umukoro, 2013; Ferreira et al., 2016) as it allows cross-country comparisons and facilitates poverty measurement, some critics point out that this approach tends to overlook “socio-historical processes of class formation” (Knauss, 2019), and determines poverty lines arbitrarily (Jayadev et al., 2015a,b). Its arguments solely rely on individualistic accounts and fail to consider historical, political, and economic transformation as well as class and power relations (Wright, 1994; Harvey and Reed, 1996).

Besides, it overlooks cultural differences and the various dynamics of different groups, which has been criticized by holders of the relativist approach (Townsend, 1979; Farah and Sampath, 1995; Erdogan, 2016; Senses, 2017; UNDP, 2019). Another criticism points out that non-monetary measures of poverty should be taken into account, such as the literacy rate, political, and social participation, etc. (UNDP, 1990, 2019; Sen, 1999). Yet another critique consists in the fact that absolute poverty approach does not consider the subjective view of the poor as claimed in the discussions on culture of poverty (although its main focus consists in examining whether there is a separate culture of poverty) started by Lewis (Lewis, 1966).^2^ Finally, an important point of contention with the absolute poverty approach is that it reduces poverty to personal income distribution tables and overlooks differences between “the poor” in various class situations and their variegated needs, such as landless peasants, the unemployed, low-paid retirees, etc. (Boratav, 2004). Critiques have called this approach to poverty “the poor detached from their societal identity” (Köse and Bahçe, 2009), as it abstracts poverty from its societal context by focusing on income distribution and poverty lines. This approach seemingly provides a clearer picture of poverty by drawing a sharp line of divide, but by doing so, it homogenizes variegated social groups and fails to provide information, such as class dynamics, power relations or simply which classes and occupations constitute the lower, “poorer” segment of society.

### Relative poverty

As a consequence of the critiques formulated against the absolute poverty approach (Townsend, 1979; Gustafsson and Lindblom, 1993; Ruggeri Laderchi et al., 2003; Bradshaw and Mayhew, 2011), the relative poverty approach emerged, which takes cultural differences into account and compares individuals and groups not only in terms of income and the maintenance of physical efficiency but also in terms of culturally specific activities and living patterns. Rather than focusing on an absolute line, it also takes into consideration distributional patterns and inequality by comparing different groups and individuals.

Townsend (1979) was the first to coin this term, and started his study with a critique of Rowntree's work: his central critique was that Rowntree's study did not consider changing customs and needs over time nor their differences between social groups. Instead, it restricts human needs to a minimal and narrow sense *via* the notion of necessary minimum nutritional intake level. Townsend's work was groundbreaking and greatly influenced all later works and perspectives on poverty, including the social exclusion, capability, and multidimensional approaches. His primary focuses are on society's role in creating and imposing different needs and wants which will be met through various resources, the importance of different styles of living, the relativity of the needs for accommodation and food, and the importance of the deprivation approach, which examines resources rather than income.

By coining the term “relative deprivation,” he argued the need to investigate the distinction between actual and socially perceived poverty, the role of society in imposing expectations, needs, and wants, as well as the interrelation between the relativity of poverty across country, culture and time on the one hand, and subsistence needs and social activities on the other (Townsend, 1979). This approach brings out the necessity to define the customs and activities that make up the “style of living” of society as well as society's resources (Townsend, 1979). He proposes two measurement tools for assessing poverty. The first one consists in the definition of all resources, “cash income, capital assets, employment benefits in kind, public social services in kind, private income in-kind,” that determine the overall standard of living in a particular society and ranking them through individual and household units (Townsend, 1979, p. 90). This shows where the deprivations lie and points to the distributional inequalities. The second measurement tool consists in an index which includes the common activities generally shared in a society that makes up the style of living and determines “a point […] below which […], families find it particularly difficult to share in the customs, activities, and diets comprising their society's style of living” (Townsend, 1979, p. 60).

This subjectivity and multidimensionality of poverty creates concern in measuring subjective indicators of poverty and in determining the indicators themselves. Townsend himself does not share a style of living indicators and points to the problem of finding “reliably represented […] indicators” for the deprivation and “style of living” approach (Townsend, 1979, p. 60).

Although Townsend's relative poverty approach is useful in so far as it shifts the attention from purely monetary concerns and takes class relations into considerations, its lack of concern for relations of exploitation and class antagonism both in the production field and in the social relations weakens the power of his theoretical approach. As Harvey points out, a similar tendency is common to other reformist and social democratic approaches: they accept that “poverty originates in class struggle but place the locus of the struggle in the domain of circulation, rather than production.” Consequently, according to this view poverty can be eliminated through distributional justice, “without actually abandoning capitalist production” (Harvey and Reed, 1996), a perspective we will criticize in the second section.

### Capability approach

Amartya Sen is the founder of the capability approach, a development theory that understands economic growth and individual income “as means to expanding freedoms” of members of society and defines development as a “process of expanding real freedoms” of individuals and as an end in itself (Sen, 1999). In this view, freedom, according to Sen, includes “capabilities” such as “avoiding starvation, premature mortality and freedoms associated with being literate, being able to participate in political and social life.” According to this view, the assessment of development should be done considering both the development of individual capabilities and the expansion of freedoms. Development also requires the removal of significant sources of unfreedom, such as poverty, according to Sen.

This approach perceives poverty as the “deprivation of basic capabilities rather than merely lowness of incomes” (Sen, 1999), and it requires defining what are the basic capabilities such as literacy, elementary healthcare, political and social participation, and analyzing the elements that prevent their development such as gender bias, race, age, and disability. This view does not deny the importance of income in poverty but emphasizes the significance of other constitutive elements of poverty, namely, “capabilities.” Hence, income is only a means for an end, which consists in freedoms and capabilities.

Despite these differences of focus, the capability approach does not radically break away from the absolute poverty approach since it underscores the “absoluteness of needs” (Sen, 1985), a central point of contention between Sen and Townsend. According to Sen, people's deprivations are to be judged in absolute terms, not in comparison with others in society, an argument founded on individualism and rooted in neoclassical economics. By contrast, Townsend considers that needs are “socially created and have to be identified and measured in that spirit” (Townsend, 1985) and points to the capability approach's limited explanatory power when it comes to “structural interrelationships” and their origins.

Indeed, Sen's *Development as Freedom* illustrates the lack of political context and overexplanatory role of individual causes in the capability approach. One of the most striking examples is Sen's analysis of success stories in Kerala, China, and Costa Rica which he compares with experiences of people in Brazil and South Africa or African-Americans in the USA, in which he finds that individuals of the former areas live longer and better lives than those in the latter. These examples are spread throughout the book:

For example, the citizens of Gabon or South Africa or Namibia or Brazil may be much richer in terms of per capita GNP than the citizens of Sri Lanka or China or the state of Kerala in India, but the latter have very substantially higher life expectancies than do the former (Sen, 1999, p. 6).

For example, in the United States, African Americans as a group have no higher—indeed have a lower—chance of reaching advanced ages than do people born in the immensely poorer economies of China or the Indian state of Kerala (or in Sri Lanka, Jamaica, or Costa Rica) …The causal influences on these contrasts (that is, between living standards judged by income per head and those judged by the ability to survive to higher ages) include *social arrangements and community relations* [emphasis added] such as medical coverage, public health care, school education, law and order, prevalence of violence and so on (Sen, 1999, p. 21–22).

Sen suggests that the reason for this discrepancy between income per capita levels and life expectancy lies within “social arrangements and community relations” such as health care and schooling. While this is true, his focus on community relations and on specific policies overlooks the overarching distinction, which is of a political and economic character. Indeed, the first cases are governed either by socialist (in China and Kerala) or social democratic governments (in Costa Rica), which surely plays a critical role in creating such a contrasted situation with, say, 1990s Brazil or South Africa. However, Sen does not make any mention of these different political situations, and terms such as capitalism or socialism rarely ever appear in his book (Navarro, 2000). Even though Sen's approach is an important attempt to change the focus from solely economic growth to non-monetary aspects of poverty in development discussions, its focus on social arrangements and policy as opposed to the broader political context, as well as power and class relations, limits its explanatory power (Navarro, 2000; Saad-Filho, 2007; Yalman, 2011).

Another issue with Sen's approach, as Navarro (2000, p. 664) points out, lies in his focus on the market as the creator not only of economic growth and progress but also of basic liberties. Sen frequently cites Adam Smith, as in the case of “freedom of exchange and transaction is itself part and parcel of the basic liberties that people have reason to value” (Sen, 1999, p. 6). He interprets the freedom to enter the market as a significant contribution to development: analyzing the exclusion of some members of society from market mechanisms as a deprivation means that the market and inclusion in the market are envisioned as a solution to poverty and the driving force of development, which is reminiscent of the social exclusion approach, discussed later.

Sen's insistence on the individual being as “the subject and object of analysis,” his overlooking of collective agents and social classes, as well as exploitation or domination in his analysis, together with his numerous references to Adam Smith point to his proximity with the classical economic tradition (Navarro, 2000). Sen's approach is also aligned with the neo-classical economic paradigm of poverty which defines “economic activity through individuals and their subjective utilities, rather than classes and their interaction” (Harvey and Reed, 1992). Sen even argues that capabilities are crucial in terms of improving the productivity and employability of the people, while referencing both Smith and Hayek:

But these capabilities are also associated with improving the productivity and employability of the people involved (expanding what is called their “human capital”) (Sen, 1999, p. 260).

All this goes to show that Sen's grounds his arguments primarily on the individual and the market, with limited political considerations, detached from analysis of class and power relations.

### UNDP's human development approach

The UNDP introduced another widely known approach based on Sen's theory: the human development approach. Sen was also among the consultants of the human development reports, whose aim was to bring back the “human dimension of development” into discussions over development and poverty (UNDP, 1990).

As indicated on their website, the UNDP's mission is the “eradication of poverty, and the reduction of inequalities and exclusion” (UNDP, 2022). In line with this aim, since the 1990s, UNDP has been publishing Human Development Reports, which point out the deficiency of development discussions that consider income and economic growth as an end in itself. Similarly to Sen, the UNDP instead considers income and economic growth as a means to reach human well-being/human development, which is the end goal of development (UNDP, 1990). The human development reports, according to UNDP, aim to lay bare the relationship between economic growth and human development and how growth helps or fails to translate into human development (UNDP, 1990, p. iii). The 1990s human development report clearly stated that high economic growth does not necessarily result in poverty reduction, as in the case of Nigeria: “rapid growth did not significantly improve the human condition” (UNDP, 1990, p. 59). Also, human development is framed as to enlarge “people's choices,” and these choices and capabilities are listed under a “human development index” (UNDP, 2019, p. 31). The Human Development Index measures “the capability to live a long and healthy life, to acquire knowledge and to earn income for a basic standard of living” along with indicators such as “life expectancy at birth, mean years of schooling, GNI per capita.” (UNDP, 2019, p. 300).

This approach has similar limitations to those associated with the capability approach. For example, one of the UNDP's strategies is the “Inclusive Markets Development Approach,” which illustrates their understanding of the market as the driver of growth and creator of human capabilities (UNDP, 2010). Similarly, their development and poverty reduction strategy consist in a “pro-poor market facilitation approach,” in which the poor's inclusion in markets is seen as a key poverty-reduction solution (UNDP, 2010, Foreword). This solution consists, in other words, in the poor's subjugation to market forces and imperatives. In this approach, poverty reduction is reduced “to market-mediated activities of buying and selling” (Harvey and Reed, 1992, p. 279) and “asset levels and skills rather than exploitative social relations” (Campling et al., 2016, p. 1,747). This approach is widely used in poverty reduction strategies.

### Social exclusion and poverty

The introduction of the concepts of poverty and social exclusion to the EU's Social Charter dates back to 1996, with the revised version of Social Charter of 1961, where “the right to protection against poverty and social exclusion” was added to article 30 (Council of Europe, 1996). In its Lisbon strategy, it then made combating social exclusion and poverty one of its strategic goals for the following decade (2000–2010), and an integral part of becoming “the most competitive and dynamic knowledge-based economy in the world capable of sustainable economic growth with more and better jobs and greater social cohesion” (Council of the European Union, 2000). In another statement, fighting against poverty and social exclusion is seen as accompanying the “modernization of the economy” (European Commission, 2004). Besides, the overall aim in fighting against poverty and social exclusion is also described as the reinforcement of “inclusiveness and cohesion of European society” and enforcement of “all citizens to enjoy equal access to opportunities and resources” (European Parliament, 2021). Poverty and social exclusion are then seen as a disruptive factor for social cohesion that needs to be eradicated.

The EU uses a “relative definition of poverty” focusing on the notions of exclusion and marginalization:

“… people are said to be living in poverty if their income and resources are so inadequate as to preclude them from having a standard of living considered acceptable in the society in which they live. Because of their poverty they may experience multiple disadvantages through unemployment, low income, poor housing, inadequate health care and barriers to lifelong learning, culture, sport and recreation. They are often *excluded and marginalized* [emphasis added] from participating in activities (economic, social and cultural) that are the norm for other people and their access to fundamental rights may be restricted.” (Eurostat, 2010)

In this context, the recommended solutions to poverty and exclusion lie in the “active inclusion” of people in the labor market and financial services (through training for skill formation, retraining, on-going job search assistance, access to financial services) along with income support/social protection services and better access to services (such as health, education) with a specific focus on “inclusion of vulnerable groups” (Eurostat, 2010).

In addition to the inclusion of individuals in the market, the EU's approach also pays attention to material inequalities and proposes income redistribution as a solution, notably *via* social protection systems such as “means-tested benefits, childcare, and tax credits” (Eurostat, 2010, p. 96). Although there is nothing wrong with supporting people through income schemes, such policies constitute mere temporary fixes which promote and accelerate the inclusion of marginalized people in capitalist society, and are bound to lead to the emergence of new issues down as long as capitalist structures are left unchallenged (Offe, 2018).

The five approaches to poverty reviewed above, despite their respective merits, share an uncritical acceptance of the current neoliberal regime together with a somewhat myopic conception of the causes of poverty, overlooking the crucial role of relations, and the antagonistic character of capitalist structures. In terms of solutions, they all foster the inclusion of outsiders into the market as the solution to poverty without questioning that very system's role in creating poverty and social exclusion in the first place. In other words, these tend to focus on proposing treating poverty's symptoms without analyzing its causes.

This tendency to ignore the causes and nature of poverty pushes such approaches to focus their effort into measuring poverty with different indicators, which define each approach. Not only does it define the approach, but it also defines poverty itself: “the measurement itself becomes a substitute for definition: to be poor is to have less than a certain income level. The poverty line, wherever it is drawn, thus defines what is poverty and who is poor” (Novak, 1995). And this measurement-focused approach detaches poverty from the working class and fosters the idea that poverty only means having less than average or quantifiable income level. In addition, the over-focus on poverty lines and nominal increases in PPP income levels obscure historical changes happening through the capitalist accumulation process, namely in capital-labor relations.

## Bringing classes back into poverty discussions

In contrast with the approaches examined above, this study proposes to study poverty from a Marxist perspective, as a byproduct of a historically determined mode of production, whose dynamics are largely funded on antagonistic class relations: surplus appropriation from one class by another constitutes a fundamental and inherent characteristic of a capitalist society founded on exploitation and class antagonism *via* the wage relation. Therefore, although poverty reduction is a critical objective, trying to abolish poverty within an economic system that rests on surplus appropriation and exploitation may be a futile effort, and any attempt at abolishing poverty should first conduct an analysis of its root causes. In this section, we develop three points that support a return of class analysis in poverty discussion.

### Poverty as inherent to capitalism

Marx argued that pauperism, “the hospital of the active labor-army and the dead weight of the industrial reserve army,” is a necessary condition for “capitalist production and capitalist development of wealth” (Marx, 1990, p. 797). The “antagonistic character of capitalist accumulation” necessitates an accumulation of misery in equal proportion with the accumulation of capital:

From day to day it thus becomes clearer that the relations of production in which the bourgeoisie moves do not have a simple, uniform character but rather a dual one; that in the same relations in which wealth is produced, poverty is produced also; that in the same relations in which there is a development of the forces of production, there is also the development of a repressive force; that these relations produce bourgeois wealth, i.e., the wealth of the bourgeois class, only by continually annihilating the wealth of the individual members of this class and by producing an ever-growing proletariat.

Therefore, what the approaches reviewed above call “social exclusion” is really the equivalent of the reserve army of labor, or the relative surplus population, something that is inherently part of capitalism, according to Marx. Historically, this claim has been largely vindicated, with the persistent presence of marginalized and impoverished segments of society, even at the height of the Fordist era and regulated capitalism in the richest capitalist countries during the decades that followed the Second World War.

In addition to protection schemes and social assistances, aimed at preventing or solving social exclusion, a lot of attention focuses on questions of access to and (re)distribution of resources, as in the case of Townsend's relative poverty and the EU's social exclusion approaches. Here, the subject of these policies includes the working poor. Overseeing the class and exploitation relations and focusing on life chances through ownership of resources projects the understanding of freedom and development, representing “present bourgeois conditions of production, free trade, free selling, and buying” (Marx and Engels, 1978).

However, many distributional solutions neglect the antagonistic character of capitalist accumulation and production relations. And distribution is a solution that cannot go beyond temporarily raising the living standards of the working class in a society based on surplus-value appropriation and the exploitation of the working class/labor force. As Boratav (1972, p. 16) puts it:

While the roles of classes in production are not changed, that is, when the production relations are fixed, attempting to arbitrarily change the amount of income (hence the distribution relations) either collapses the production or the basic economic laws on distribution render such measures ineffective. Old distribution relations prevail after an adjustment period.

### Uncritical acceptance of the neoliberal regime

One of the characteristics of the mainstream approaches on poverty reviewed above is their tendencies to naturalize the current economic regime and its relations of production. Notably, these perspectives do not openly question the current global neoliberal regime, which, following the “internationalization of policy regimes” (Jessop, 2002) have been led and promoted by the IMF and the World Bank, key “trans-national regulatory agencies” that introduced neoliberal regime as “model of development” (Neilson, 2020). The policies instructed within this regime *via* Structural Adjustment Programs (SAP) consist of trade and financial liberalization, privatizations, labor market flexibilization, deregulation, and promotion of the market economy, which increases workers' exploitation, and favors the accumulation of capital and the reimbursement of public debt over social services and redistribution of income, eventually exacerbating issues of poverty.

Despite its poverty reduction objectives, the World Bank's programs “were conditional upon satisfactory macroeconomic and sector policy indicators” of the IMF's SAP (World Bank, 2000). This process led to the rise of the hegemony of international financial organizations in the area of “development,” which came to be reduced to liberalizations and global market integration, in parallel with poverty reduction programs. This approach contrasts with the understanding of previous decades when development was seen and implemented through employment policies, public investment, and industrialization (Rowden, 2010). These policies together with poverty reduction programs, led to the global diffusion of capitalism's “unstable logic of accumulation,” leading to both the subordination of nation-states to the needs of capital (Neilson, 2020) and the reconstitution of “capitalist class supremacy” within the nation-state (Saad-Filho, 2019).

The strategy of the “inclusion” of the “outsiders” to the neoliberal system and its market forces leads to the subordination of labor to capital *via* processes of dispossession, depeasantization and proletarianization, as examined in the next section. This uncritical acceptance of the current neoliberal system and policies results in treating poverty as an unrelated problem with its own external causes (psychological, cultural, geographical, etc.). Instead, by disregarding class dynamics and emphasizing the success stories of poverty reduction (with poverty line analyses), these approaches legitimize the neoliberal and capitalist system, whereas we claim that they are one of the key factors in poverty today.

### What do poverty thresholds mean?

First of all, poverty thresholds are set arbitrarily, and the cutting line between poor and non-poor, or between poor and middle-class, has been the topic of long debates. The threshold of $1.9 PPP per person and per day has been used by the World Bank as the cut-off line for global poverty. In the literature, thresholds of $2 PPP or even $10 PPP have been used. Given that a variation of a few cents in the cut-off line is bound to shift the category of tens of millions of people from “poor” to “non-poor” (or vice versa), the poverty threshold method to understanding and measuring poverty is inherently approximate.

Adding to this, PPP calculations, although they constitute the best tool available for international income comparisons, are themselves inherently approximate by nature. Similar to the threshold, a slight change in PPP calculation is likely to result in radically different poverty statistics. Finally, even if one were to take PPP threshold statistics as face value, what does it concretely mean for people to surpass the $1.90 threshold and reach a $2 per day income, for example? Would these individual even be aware that they have been “lifted from poverty”? We would suggest that such absolute poverty thresholds, in a context of high GDP growth in a number of low income countries over the last 20 years, lead to spectacular statistical results that make the headlines of World Bank reports (“Hundreds of millions of people lifted out of poverty”) but actually have limited equivalence in concrete material conditions (Knauss, 2019).^3^

As a consequence, it would be advisable to avoid analyzing poverty solely through the lens of PPP thresholds, as the World Bank and its absolute poverty approach does, and adopt instead a broader understanding that takes class dynamics into account, thereby providing explanations for the underlying social and economic transformations that get reflected in statistical evidence.

### Poverty lines and the mystification of class dynamics

Finally, adopting a Marxist analysis of poverty would allow us to understand the historical dynamics that are at play behind statistical moves below and above poverty thresholds. The excessive reliance on poverty thresholds and nominal rises in PPP income levels obscures structural transformations resulting from capitalist dynamics. We want to emphasize two aspects in particular.

The first aspect is the effect of East and South-East Asia on global poverty statistics. Both of these regions have experienced a particularly robust economic growth over the last two decades, leading to a significant rise in per capita PPP daily income. The weight of China, in particular, in the global population, means that successful “poverty eradication” there (with a staggering collapse of extreme poverty, from 92% of Chinese population in 1981 down to 0.43% by 2017), translates as hundreds of millions of people passing above the $1.9 PPP threshold. While this is *a priori* a success story for China and its working class, it can easily be interpreted as a success for the whole world, whereas one does not observe much, if any, poverty reduction (as measured by the $1.9 PPP threshold) in other regions of the world. Therefore, one should be cautious with the triumphalist prediction on the end of global poverty and lead a regional analysis that considers the essential role of East and South-East Asia in global commodity chains, and the effect it had on the working classes of these regions compared to the rest of the world.

The second and most important aspect is that the poverty threshold approaches obscure the class-based dynamics and historical transformations that are at play. Most notably, capital accumulation through dispossession, depeasantization and proletarianization is arguably one of the key processes that led to the rise of daily PPP income. The massive wave of depeasantization observed in the last 20 years as a consequence of neoliberal policies forcing peasants off their land, resulted in processes of proletarianization and monetization of income, which pushed income levels upwards without this necessarily corresponding to actual improvements in conditions of living. This global wave of proletarianization, happens to correlate neatly with:

the major fall in the portion of humanity under $2 PPP per day, or what is known as “poverty reduction” among the policy institutions (Knauss, 2019).

This suggests that the current progress in poverty reduction observed with mainstream measurements, is in fact nothing but the reflection of a historical process of dispossession, depeasantization, and proletarianization whereby dispossession is done through dispossessing the means of production of producers, which also includes dispossession in the rural areas where the rural dwellers are being pushed out of land and, in some cases, proletarianized, when they do not enter the informal sector. This historical process is called primitive accumulation by Marx which creates, *continually reinforces and extend the scale of* ^4^ capitalist relations, classes, and exploitation, in favor of capital. Consequently, such improvement in per capita PPP income may indeed be a one-time phenomenon, in which case the trends currently observed in global poverty reduction may quickly come to an end (Knauss, 2019). This shows both the limitations of mainstream approaches and measurements of poverty, and the importance of analyzing poverty through a Marxist lens that allows us to understand the transformations of social and economic relations that result from capitalist dynamics.

## Conclusion

In this article, we argued that understanding poverty requires examining the causes and forces that drive social and historical changes, particularly as they relate to the antagonistic class relations that characterize the capitalist mode of production. As studied above, the mainstream literature on poverty centers its analysis on abstract poverty lines, PPP income numbers, or poverty's relative and exclusionary aspects. While such approaches provide measurements of social and economic reality that can be useful for analysis, they also miss important dynamics by abstracting the class positions of the poor and homogenizing society *via* individual income and consumption calculations. The related discussions over social inclusion and exclusion foster the acceptance, given minor fixes, of the current global neoliberal regime without questioning its role in creating and exacerbating the very phenomenon they propose to eradicate, namely poverty. Ignoring class relations and exploitation by focusing on life chances, skills and assets legitimize, and naturalizes the present bourgeoisie conditions of production. Debates on poverty also tend to overlook the working class, and act as if poor people were a separate class and social group that is not inserted within capital-labor relations. These mainstream approaches to poverty allow poverty analysts and policymakers to move in a “hegemonically safe space” that ignores perspectives that would require structural changes in wealth, power, and the capitalist system itself (Harvey and Reed, 1992). We would therefore recommend bringing class and socio-historical analyses back in poverty discussion, thereby delivering more accurate depictions of social reality based on class relations, on which to design an effective solution to global poverty eradication.

## Author contributions

GÖ conducted the literature review, developed the initial idea of the paper, and wrote the first draft of the paper under the guidance of AD. The article was then revised by GÖ and AD. Both authors contributed to the article and approved the submitted version.

## Footnotes

^1^One of the central limits of measurement at household level being that it overlooks gender inequality.

^2^Lewis's culture of poverty approach is a highly debated approach. On the one side, it is debated that the theory is based on Marxist critique of capital and its contradictions and highlights the resilience and coping mechanisms of the poor (Harvey and Reed, 1996); on the other side it is argued that the culture of poverty “blames the poor as victim” (Valentine, 1969) through their lack of ability and portrays them negatively (Stack, 1974, as cited in Harvey and Reed, 1996).

^3^In 2017, 9.61% of the world population (i.e., 730 m people) lived with < $2.15 per day, according to World Bank (Poverty and Inequality Platform).

^4^Even though Marx defines this process as “primitive” accumulation and refers to it as the pre-history of capital, he also emphasizes the continuation and maintenance of this separation “on a constantly extending scale” (Marx, 1990, p. 874). Through highlighting the continuity of the process, Harvey (2003) later theorizes it as “accumulation by dispossession”.

## References

[B1] Boratav K. (2004). “Yoksulluk” kavrami üzerine notlar. Toplum ve Hekim 19, 7–9.

[B2] Bradshaw J. Mayhew E. (2011). The Measurement of Extreme Poverty in the European Union. DG Employment, Social Affairs and Inclusion: European Commission.

[B3] Campling L. Miyamura S. Pattenden J. Selwyn B. (2016). Class dynamics of development: A methodological note. Third World Q. 37, 1745–1767.

[B4] Council of Europe (1996). European Social Charter (Revised). ETS 163. Available online at: https://www.refworld.org/docid/3ae6b3678.html (accessed January 5, 2023).

[B5] Council of the European Union (2000). Presidency Conclusions. Lisbon: Council of the European Union.

[B6] Erdogan N. (2016). Yoksulluk Halleri. Iletisim. Available online at: https://iletisim.com.tr/kitap/yoksulluk-halleri/8041 (accessed June 6, 2022).

[B7] European Commission (2004). Joint Report on Social Inclusion. European Commission Directorate-General for Employment and Social Affairs.

[B8] European Parliament (2021). The Fight Against Poverty, Social Exclusion and Discrimination. European Parliament.

[B9] Eurostat (2010). Combating Poverty and Social Exclusion. A Statistical portrait of the European Union. Eurostat Statistical Books.

[B10] Farah A. A. M. Sampath R. K. (1995). Poverty in Sudan. J. Asian Afr. Stud. 30, 146–161. 10.1177/002190969503000302

[B11] Ferreira F. H. Chen S. Dabalen A. Dikhanov Y. Hamadeh N. Jolliffe D.. (2015). A Global Count of the Extreme Poor in 2012: Data Issues, Methodology and Initial Results, Working Paper. Washington, DC: World Bank. 10.1596/1813-9450-7432

[B12] Ferreira F. H. Chen S. Dabalen A. Dikhanov Y. Hamadeh N. Jolliffe D.. (2016). A global count of the extreme poor in 2012: data issues, methodology and initial results. J. Econ. Inequal. 14, 141–172. 10.1007/s10888-016-9326-6

[B13] Gustafsson B. Lindblom M. (1993). Poverty lines and poverty in seven European countries, Australia, Canada and the USA. J. Euro. Soc. Policy 3, 21–38. 10.1177/095892879300300102

[B14] Harvey D. L. (2003). The New Imperialism. Oxford: Oxford University Press. 10.1093/oso/9780199264315.001.0001

[B15] Harvey D. L. Reed M. H. (1992). Paradigms of povert: a critical assessment of contemporary perspectives. Int. J. Polit. Cult. Soc. 6, 269–297. 10.1007/BF01395301

[B16] Harvey D. L. Reed M. H. (1996). The culture of poverty: an ideological analysis. Sociol. Perspect. 39, 465–495. 10.2307/1389418

[B17] Jayadev A. Lahoti R. Reddy Sanjay G. (2015b). Who Got What, Then and Now? A Fifty Year Overview from the Global Consumption and Income Project, Working Papers. 2015_04. University of Massachusetts Boston, Economics Department. Available online at: https://ideas.repec.org/p/mab/wpaper/2015_04.html (accessed June 6, 2022).

[B18] Jayadev A. Lahoti R. Reddy Sanjay G. (2015a). The Middle Muddle: Conceptualizing and Measuring the Global Middle Class. 10.2139/ssrn.2694624

[B19] Jessop B. (2002). Liberalism, neoliberalism, and urban governance: a state–theoretical perspective. Antipode 34, 452–472. 10.1111/1467-8330.00250

[B20] Knauss S. (2019). The myth of the global middle class, globalisation's fallback success story. Can. J. Dev. Stud. 40, 182–200. 10.1080/02255189.2019.1520692

[B21] Köse A. H. Bahçe S. (2009). “Yoksulluk” yazininin yoksullugu: toplumsal siniflarla düşünmek. Praksis 19, 385–419.

[B22] Lewis O. (1966). The culture of poverty. Sci. Am. 215, 19–25. 10.1038/scientificamerican1066-19 5916451

[B23] Marx K. (1990). Capital: A Critique of Political Economy, Vol. 1, ed B. Fowkes. London: Penguin Books.

[B24] Marx K. Engels F. (1978). The Marx-Engels Reader, 2nd Revised & enlarged edition, ed R. C. Tucker. New York, NY: W.W. Norton and Company.

[B25] Navarro V. (2000). Development and quality of life: a critique of amartya sen's development as freedom. Int. J. Health Serv. 30, 661–674. 10.2190/10XK-UYUC-E9P1-CLFX 11127016

[B26] Neilson D. (2020). Bringing in the “neoliberal model of development.” Cap. Class 44, 85–108. 10.1177/0309816819852746

[B27] Novak T. (1995). Rethinking poverty. Crit. Soc. Policy 15, 58–74. 10.1177/026101839501504404

[B28] Offe C. (2018). Contradictions of the Welfare State. Routledge. 10.4324/9780429464829

[B29] Ravallion M. Datt G. van de Walle D. (1991). Quantifying absolute poverty in the developing world. Rev. Income Wealth 37, 345–361. 10.1111/j.1475-4991.1991.tb00378.x

[B30] Rowden R. (2010). Poverty reduction is not development. Rev. Afr. Polit. Econ. 37, 503–516. 10.1080/03056244.2010.530949

[B31] Rowntree B. S. (1902). Poverty: A Study of Town Life, 2nd Edn. London: Macmillan.

[B32] Ruggeri Laderchi C. Saith R. Stewart F. (2003). Does it matter that we do not agree on the definition of poverty? A comparison of four approaches. Oxford Dev. Stud. 31, 243–274. 10.1080/1360081032000111698

[B33] Saad-Filho A. (2007). Life beyond the washington consensus: an introduction to pro-poor macroeconomic policies. Rev. Polit. Econ. 19, 513–537. 10.1080/09538250701622352

[B34] Saad-Filho A. (2019). Democracy Against Neoliberalism: The Limitations of Neoliberal Democracy in Value and Crisis: Essays on Labour, Money and Contemporary Capitalism. Brill Publisher. 10.1163/9789004393202

[B35] Sen A. (1985). A sociological approach to the measurement of poverty: a reply to professor peter townsend. Oxford Econ. Pap. 37, 669–676. 10.1093/oxfordjournals.oep.a041716

[B36] Sen A. (1999). Development as Freedom. New York, NY: Alfred Knopf.

[B37] Senses F. (2017). Küreselleşmenin Öteki Yüzü Yoksulluk. Istanbul: Iletişim Yayinlari.

[B38] Townsend P. (1979). Poverty in the United Kingdom: A Survey of Household Resources and Standards of Living. Berkeley, CA; Los Angeles, CA: University of California Press. 10.1525/9780520325760

[B39] Townsend P. (1985). A sociological approach to the measurement of poverty—a rejoinder to professor Amartya Sen. Oxford Econ. Pap. 37, 659–668. 10.1093/oxfordjournals.oep.a041715

[B40] Umukoro N. (2013). Poverty and social protection in Nigeria. J. Dev. Soc. 29, 305–322. 10.1177/0169796X13494281

[B41] UNDP (1990). Human Development Report 1990: Concept and Measurement of Human Development. New York, NY: UNDP.

[B42] UNDP (2019). Human Development Report 2019. Beyond Income, Beyond Averages, Beyond Today: Inequalities in Human Development in the 21st Century. New York, NY: UNDP.

[B43] UNDP (2022). About Us. United Nations Development Programme, UNDP. Available online at: https://www.undp.org/about-us (accessed June 11, 2022).

[B44] UNDP. (2010). Inclusive Markets Development Handbook. New York, NY: Private Sector Division Partnerships Bureau, UNDP.

[B45] Valentine C. A. (1969). Culture and poverty: critique and counter-proposals. Curr. Anthropol. 10, 181–201. 10.1086/201071

[B46] World Bank (1990). World Development Report 1990: Poverty. Washington, DC: World Bank. 10.1596/0-1952-0851-X

[B47] World Bank (2000). Turkey—Country Assistance Strategy (English). Washington, DC: World Bank. Available online at: http://documents.worldbank.org/curated/en/888881468760497558/Turkey-Country-assistance-strategy (accessed January 5, 2023).

[B48] World Bank (2021). *The International Poverty Line has Just Been Raised to $1.90 a Day, But Global Poverty is Basically Unchanged*. How Is That Even Possible? Washington, DC: World Bank.

[B49] Wright E. O. O. (1994). Interrogating Inequality: Essays on Class Analysis, Socialism and Marxism. London; New York, NY: Verso Books.

[B50] Yalman G. (2011). “Discourse and practice of poverty reduction strategies: reflections on the Turkish case in the 2000s,” in Converging Europe, ed I. Eren (London: Ashgate).

